# Magnetic Resonance Cholangiopancreatography-Derived Anatomic Predictors of Difficult Biliary Cannulation During Endoscopic Retrograde Cholangiopancreatography: A Retrospective Cohort Study

**DOI:** 10.7759/cureus.110710

**Published:** 2026-06-12

**Authors:** Antony Kahwagi, Perla Wehbe, Hassan Mohamed Ali, Maria Njeim, Eid Mahfouz, Joseph Amara, Khalil Honein, Rita Slim, Cesar Yaghi

**Affiliations:** 1 Gastroenterology and Hepatology, Hôtel-Dieu de France, Beirut, LBN; 2 Radiology, Hôtel-Dieu de France, Beirut, LBN

**Keywords:** choledochoduodenal angle, common bile duct, difficult biliary cannulation, endoscopic retrograde cholangiopancreatography, magnetic resonance cholangiopancreatography, periampullary diverticulum

## Abstract

Background

Difficult biliary cannulation during endoscopic retrograde cholangiopancreatography (ERCP) is associated with procedural failure, prolonged manipulation of the papilla, and a higher risk of post-ERCP adverse events. Pre-procedural magnetic resonance cholangiopancreatography (MRCP) may provide anatomic markers that help anticipate technical difficulty before ERCP.

Objective

The main objective of this study is to assess whether MRCP-derived anatomic variables predict difficult biliary cannulation according to European Society of Gastrointestinal Endoscopy (ESGE) criteria, to evaluate their association with the burden of difficult cannulation, and to explore the relationship between difficult cannulation and ERCP failure.

Methods

We retrospectively analyzed 121 consecutive patients who underwent contrast-enhanced MRCP followed by ERCP. Multiplanar 3D MRCP reconstructions were reviewed by a radiologist blinded to ERCP outcomes. On reconstructed coronal images, the choledochoduodenal angle was measured between the distal common bile duct axis and the adjacent duodenal tangent at the point of biliary termination, while the distal common bile duct diameter was measured using an outer wall-to-outer wall technique within 2 cm proximal to the major papilla; papillary bulging suggestive of periampullary diverticulum was recorded. Difficult cannulation was defined by at least one ESGE criterion: more than five contacts with the papilla, more than five minutes spent attempting to cannulate after papillary visualization, or more than one unintended pancreatic duct cannulation or opacification. Multivariable logistic regression was used for global difficult cannulation and for individual ESGE criteria. Cumulative logit ordinal regression was used to model the number of ESGE criteria met. Because ERCP failure was infrequent, Firth penalized logistic regression was used for failure analyses.

Results

Difficult cannulation was present in 82/121 patients (67.8%), and ERCP technical success was achieved in 111/121 patients (91.7%). In multivariable analysis, a smaller distal common bile duct diameter independently predicted global difficult cannulation (OR 0.899, 95% CI 0.810-0.999; p = 0.047). For the individual ESGE criteria, a narrower choledochoduodenal angle (OR 0.977, 95% CI 0.955-0.999; p = 0.038) and a smaller distal common bile duct diameter (OR 0.884, 95% CI 0.794-0.984; p = 0.024) were independently associated with more than five papillary contacts, while a narrower choledochoduodenal angle was also associated with cannulation time greater than five minutes (OR 0.978, 95% CI 0.9573-0.9999; p = 0.049). In ordinal regression, both a narrower choledochoduodenal angle (OR 0.980, 95% CI 0.961-0.999; p = 0.040) and a smaller distal common bile duct diameter (OR 0.892, 95% CI 0.811-0.981; p = 0.019) were associated with a higher burden of difficult cannulation. MRCP anatomy alone did not predict ERCP failure. However, all failures occurred in patients with difficult cannulation, and each additional ESGE criterion increased the odds of ERCP failure by 3.66-fold (95% CI 1.67-11.39; p = 0.0003).

Conclusions

MRCP-derived anatomy, particularly distal common bile duct diameter and choledochoduodenal angulation, is associated with difficult ERCP cannulation and with a greater burden of cannulation difficulty. Final ERCP failure appears to be driven more strongly by the cumulative burden of difficult cannulation than by MRCP anatomy alone.

## Introduction

Selective biliary cannulation is the critical first step in therapeutic endoscopic retrograde cholangiopancreatography (ERCP). When biliary access is not achieved promptly, the procedure becomes longer, technically more demanding, and more likely to require advanced rescue maneuvers. Difficult access is also clinically important because repeated papillary trauma and prolonged manipulation are closely linked to post-ERCP adverse events, especially post-ERCP pancreatitis [1-7].

To standardize the concept of difficult biliary access, the European Society of Gastrointestinal Endoscopy (ESGE) defined difficult cannulation as the presence of at least one of the following: more than five contacts with the papilla whilst attempting to cannulate, more than five minutes spent attempting to cannulate after visualization of the papilla, or more than one unintended pancreatic duct cannulation or opacification [1]. Beyond standardizing terminology, this definition has direct clinical relevance because it helps determine when repeated standard attempts should stop, when rescue access should begin, and how preventive measures should be tailored during ERCP [8-14]. International consensus recommendations and meta-analyses have increasingly supported earlier precut or alternative access strategies once difficult access criteria accumulate, rather than persisting indefinitely with repeated standard attempts [6,10-14].

Difficult cannulation is multifactorial. Large multicenter studies and tertiary center series have identified the influence of papillary morphology, periampullary diverticulum, altered anatomy, distal malignant obstruction, inflammatory distortion, and operator-related factors on both cannulation success and complications [8-10,15-23]. More recently, formal prediction efforts have attempted to transform these procedural and anatomical variables into practical risk stratification tools for difficult biliary access [24].

Among these predictors, periampullary diverticulum deserves particular nuance. Older single-center studies reported conflicting effects on cannulation success [18], whereas more recent reviews and meta-analytic data suggest that the impact of diverticula depends on the relationship between the papilla and the diverticulum, the underlying indication, and operator experience [19,25,26]. Likewise, the risk profile of difficult cannulation is not identical across benign stone disease, inflammatory presentations, and malignant distal obstruction [21,22]. This variability helps explain why a purely endoscopic prediction approach may be incomplete.

Cross-sectional imaging is attractive in this setting because it is available before ERCP and may capture patient-specific anatomy that cannot be fully appreciated by endoscopic inspection alone. Although computed tomography has already been used to identify anatomical predictors of difficult cannulation, magnetic resonance cholangiopancreatography (MRCP) offers several complementary advantages for evaluating biliary anatomy, including radiation-free duct-focused imaging, high contrast resolution of the biliary tree, and multiplanar visualization of the distal common bile duct in relation to the adjacent duodenum. In addition, MRCP is already incorporated into the routine diagnostic pathway of many patients with suspected biliary obstruction or choledocholithiasis, making it a clinically practical modality for pre-procedural risk stratification. Lee et al. [27] showed on computed tomography that a narrower choledochoduodenal angle and a smaller distal common bile duct diameter were associated with difficult cannulation, while endoscopic ultrasound-based data have likewise suggested that pre-procedural duct-axis relationships may help predict difficult biliary access [28]. However, MRCP-based anatomical predictors of difficult cannulation remain insufficiently characterized.

The primary objective of this study was to evaluate the association between MRCP-derived anatomical variables, specifically distal common bile duct diameter, choledochoduodenal angle, and periampullary diverticulum, and global difficult biliary cannulation as defined by the ESGE criteria. Secondary analyses examined the association of these anatomical variables with each individual difficult cannulation criterion and with the cumulative burden of difficult cannulation, expressed as the number of criteria met. An exploratory, hypothesis-generating analysis then assessed whether the cumulative burden of difficult cannulation was associated with final ERCP failure.

## Materials and methods

Study design and population

This was a single-center retrospective cohort study conducted at Hôtel-Dieu de France, Beirut, Lebanon.

We included consecutive patients, evaluated between June 2022 and October 2025, who underwent contrast-enhanced MRCP followed by ERCP and for whom both the MRCP images and the ERCP report were available for review. The study cohort, therefore, represented a paired MRCP-ERCP population rather than all-comers undergoing ERCP.

Inclusion criteria were: (1) available pre-procedural MRCP suitable for anatomical measurement; and (2) sufficiently detailed ERCP documentation to assess all ESGE difficult cannulation criteria. Exclusion criteria were: (1) no pre-procedural MRCP; (2) non-diagnostic or incomplete MRCP precluding standardized measurement of the study variables; and (3) incomplete ERCP documentation preventing reliable ascertainment of the difficult cannulation criteria.

ERCP indications were retrospectively retrieved from the institutional ERCP database.

MRCP variables

The MRCP-derived variables were selected a priori on the basis of anatomical plausibility and prior imaging literature [27]. They included the choledochoduodenal angle, distal common bile duct diameter, and periampullary diverticulum.

Multiplanar reconstruction was used to obtain a plane in which the distal common bile duct and adjacent duodenal wall could be assessed together. Measurements were performed by a single radiologist who was blinded to ERCP outcomes. The choledochoduodenal angle was defined as the angle between the distal common bile duct axis and the tangent to the adjacent duodenal wall at the point of biliary termination. Distal common bile duct diameter was defined as the maximal transverse outer-wall-to-outer-wall diameter measured within 2 cm proximal to the major papilla. Periampullary diverticulum was recorded as present or absent.

ERCP outcomes

The primary endpoint was difficult cannulation according to ESGE criteria [1]. Difficult cannulation was considered present when at least one of the following occurred: more than five contacts with the papilla whilst attempting to cannulate, more than five minutes spent attempting to cannulate after papillary visualization, or more than one unintended pancreatic duct cannulation or opacification.

Secondary endpoints were each individual ESGE criterion, the cumulative number of ESGE criteria met (zero, one, two, or three), and ERCP failure. Technical success was defined as the absence of final ERCP failure and was derived directly from the recorded failure variable.

All ERCP procedures were performed by expert endoscopists using a Pentax ED-3490TK duodenoscope (PENTAX Medical, Tokyo, Japan) and a COOK sphincterotome (G25227; Cook Medical Europe, Limerick, Ireland).

Statistical analysis

Continuous variables were summarized as medians with interquartile ranges (IQRs), and categorical variables as counts and percentages.

The three MRCP variables were retained a priori in all primary multivariable models, irrespective of univariable significance. Global difficult cannulation and each individual ESGE criterion were analyzed using multivariable logistic regression. Odds ratios were expressed per 1-degree increase in choledochoduodenal angle and per 1-mm increase in distal common bile duct diameter.

The burden of difficult cannulation, defined as the number of ESGE criteria met (zero to three), was modeled using cumulative logit ordinal regression. Because only 10 ERCP failures occurred, Firth penalized logistic regression was used for failure analyses to reduce small-sample bias [11,12]. Fisher's exact testing was used to analyze the association between difficult cannulation outcomes and final ERCP failure.

Each model used complete-case analysis for the variables required by that model. Two-sided p-values below 0.05 were considered statistically significant. The primary focus of the analysis was estimation and clinical interpretation of the MRCP-anatomy effect sizes rather than discrimination alone.

## Results

Patient characteristics

The cohort included 121 patients. Median age was 70.0 years (IQR, 59.0-83.0), and among the 115 patients with recorded sex, 63 (54.8%) were male. Among available baseline clinical variables, hypertension was present in 65/115 patients (56.5%), diabetes in 36/115 (31.3%), dyslipidemia in 55/115 (47.8%), current or former smoking in 34/115 (29.6%), and previous sphincterotomy in 19/117 (16.2%). Median choledochoduodenal angle was 45.0° (IQR, 35.0°-60.0°), median distal common bile duct diameter was 5.0 mm (IQR, 3.0-7.0 mm), and periampullary diverticulum was present in 7/121 patients (5.8%). Baseline characteristics and procedural outcomes are summarized in Table 1.

**Table 1:** Baseline clinical characteristics, ERCP indication categories, MRCP measurements, and procedural outcomes ERCP: endoscopic retrograde cholangiopancreatography; MRCP: magnetic resonance cholangiopancreatography; ESGE: European Society of Gastrointestinal Endoscopy; IQR: interquartile range

Variable	Value
Baseline characteristics	
Age, years	70.0 (59.0-83.0)
Male sex	63/115 (54.8%)
Female sex	52/115 (45.2%)
Hypertension	65/115 (56.5%)
Diabetes mellitus	36/115 (31.3%)
Dyslipidemia	55/115 (47.8%)
Current/former smoking	34/115 (29.6%)
Previous sphincterotomy	19/117 (16.2%)
ERCP indication categories	
Stone-related biliary disease	84/121 (69.4%)
Malignant obstruction/stricture	21/121 (17.4%)
Cholangitis/infectious cholestasis	8/121 (6.6%)
Stent-related reintervention	4/121 (3.3%)
Postoperative bile leak/collection	2/121 (1.7%)
Other/indeterminate benign presentation	2/121 (1.7%)
MRCP anatomical variables	
Choledochoduodenal angle, degrees	45.0 (35.0-60.0)
Distal common bile duct diameter, mm	5.0 (3.0-7.0)
Periampullary diverticulum	7/121 (5.8%)
Cannulation outcomes	
Global difficult cannulation	82/121 (67.8%)
>5 papillary contacts	69/121 (57.0%)
>5 minutes to cannulate	65/121 (53.7%)
>1 unintended pancreatic duct cannulation/opacification	36/121 (29.8%)
ERCP technical success	111/121 (91.7%)
ERCP failure	10/121 (8.3%)

**Figure 1:**
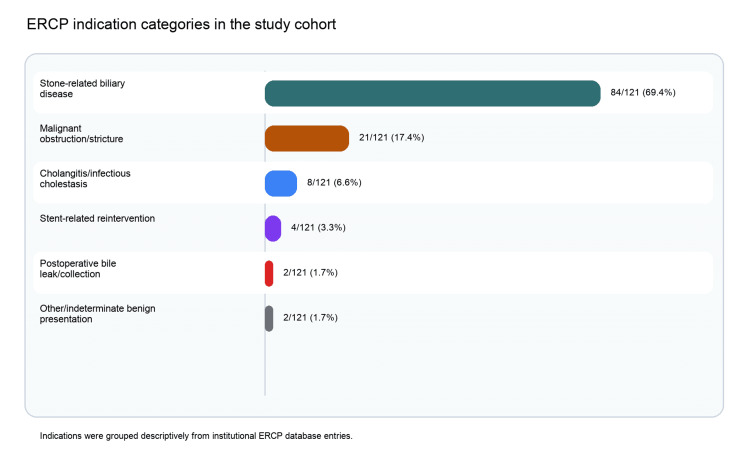
Distribution of ERCP indication categories in the study cohort ERCP: endoscopic retrograde cholangiopancreatography

The leading ERCP indication was stone-related biliary disease in 84/121 patients (69.4%), followed by malignant obstruction or stricture in 21/121 (17.4%), cholangitis or infectious cholestasis in 8/121 (6.6%), stent-related reintervention in 4/121 (3.3%), and postoperative bile leak or collection in 2/121 (1.7%); two additional patients (1.7%) had other or indeterminate benign presentations. These indication categories are listed in Table 1 and displayed in Figure 1.

Global difficult cannulation according to ESGE criteria occurred in 82/121 patients (67.8%). The individual difficult cannulation criteria were met in 69/121 patients (57.0%) for more than five papillary contacts, 65/121 (53.7%) for cannulation time greater than five minutes, and 36/121 (29.8%) for more than one unintended pancreatic duct cannulation or opacification. The burden of difficult cannulation was zero criteria in 39 patients (32.2%), one criterion in 20 (16.5%), two criteria in 36 (29.8%), and all three criteria in 26 (21.5%). ERCP technical success was achieved in 111/121 procedures (91.7%), while final ERCP failure occurred in 10/121 (8.3%). These frequencies are also shown in Table 1.

MRCP predictors of global difficult cannulation

In multivariable logistic regression, a smaller distal common bile duct diameter independently predicted global difficult cannulation (OR 0.899, 95% CI 0.810-0.999; p = 0.047). Choledochoduodenal angle showed the same protective direction but did not reach statistical significance (OR 0.982, 95% CI 0.960-1.004; p = 0.115). Periampullary diverticulum was not associated with global difficult cannulation (OR 1.112, 95% CI 0.203-6.103; p = 0.902). The multivariable estimates for global difficult cannulation and cumulative difficulty burden are summarized in Table 2.

**Table 2:** Multivariable MRCP predictors of global difficult cannulation and cumulative difficulty burden MRCP: magnetic resonance cholangiopancreatography; OR: odds ratio; CI: confidence interval; ESGE: European Society of Gastrointestinal Endoscopy

Outcome	Predictor	Adjusted OR (95% CI)	p-value
Global difficult cannulation	Choledochoduodenal angle	0.982 (0.960-1.004)	0.115
Global difficult cannulation	Distal common bile duct diameter	0.899 (0.810-0.999)	0.047
Global difficult cannulation	Periampullary diverticulum	1.112 (0.203-6.103)	0.902
Difficulty burden (0-3 ESGE criteria)	Choledochoduodenal angle	0.980 (0.961-0.999)	0.040
Difficulty burden (0-3 ESGE criteria)	Distal common bile duct diameter	0.892 (0.811-0.981)	0.019
Difficulty burden (0-3 ESGE criteria)	Periampullary diverticulum	1.227 (0.278-5.407)	0.787

MRCP predictors of the individual ESGE criteria

**Figure 2:**
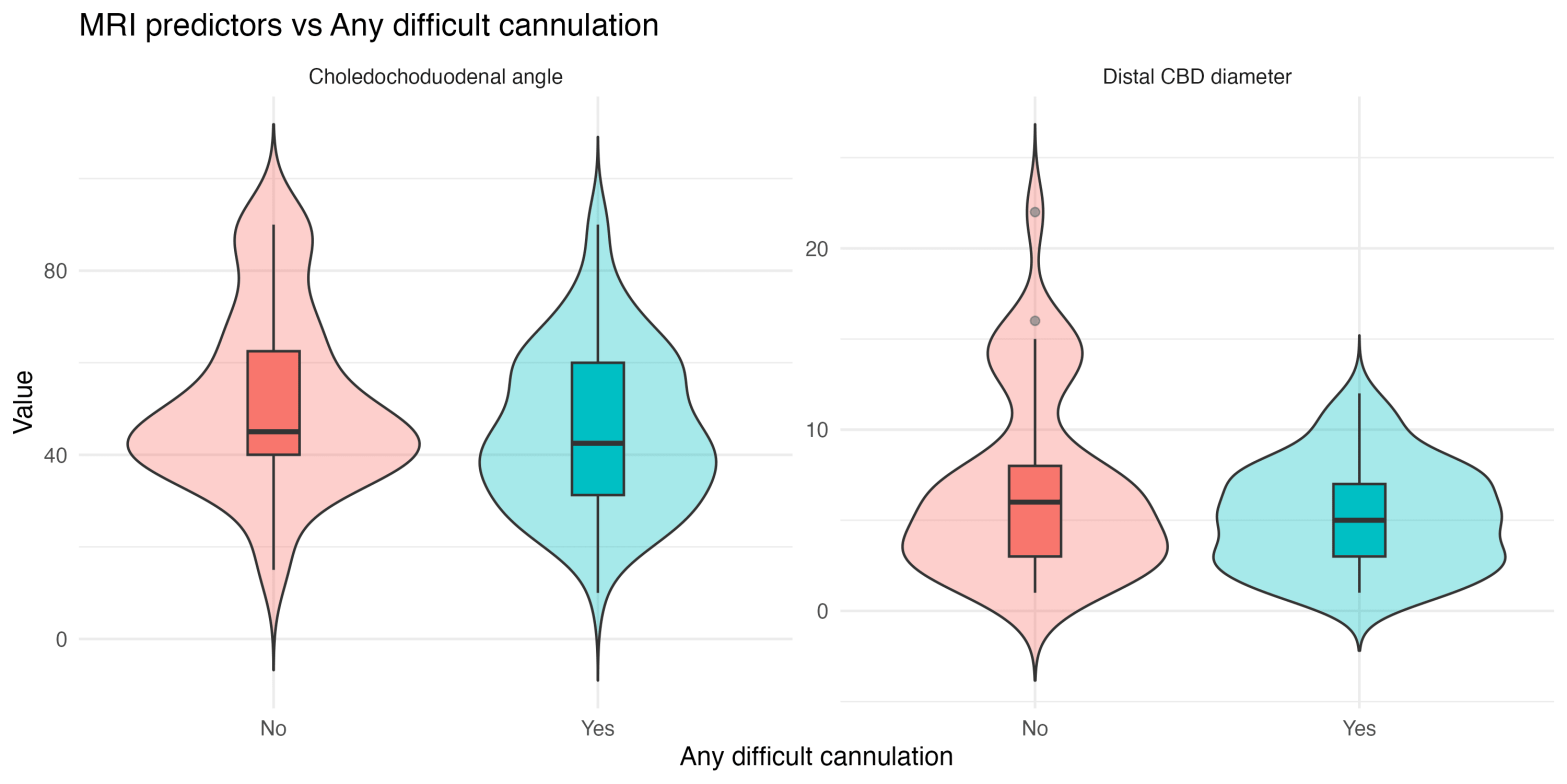
Distribution of the main MRCP anatomical predictors according to difficult cannulation status MRI: magnetic resonance imaging; CBD: common bile duct; MRCP: magnetic resonance cholangiopancreatography

For the ESGE criterion of more than five contacts with the papilla whilst attempting to cannulate, both a narrower choledochoduodenal angle (OR 0.977, 95% CI 0.955-0.999; p = 0.038) and a smaller distal common bile duct diameter (OR 0.884, 95% CI 0.794-0.984; p = 0.024) were independently associated with increased difficulty. For the criterion of cannulation time greater than five minutes, a narrower choledochoduodenal angle remained independently associated with difficulty (OR 0.978, 95% CI 0.9573-0.9999; p = 0.049), whereas distal common bile duct diameter and periampullary diverticulum were not significant. For the criterion of more than one unintended pancreatic duct cannulation or opacification, none of the MRCP variables were significantly associated with the outcome. Criterion-specific multivariable associations are shown in Table 3, and the distributions of the two main anatomical predictors by difficult cannulation status are illustrated in Figure 2.

**Table 3:** Multivariable MRCP predictors of the individual ESGE difficult cannulation criteria MRCP: magnetic resonance cholangiopancreatography; OR: odds ratio; CI: confidence interval; ESGE: European Society of Gastrointestinal Endoscopy

Outcome	Predictor	Adjusted OR (95% CI)	p-value
>5 papillary contacts	Choledochoduodenal angle	0.977 (0.955-0.999)	0.038
>5 papillary contacts	Distal common bile duct diameter	0.884 (0.794-0.984)	0.024
>5 papillary contacts	Periampullary diverticulum	1.065 (0.193-5.860)	0.943
>5 minutes to cannulate	Choledochoduodenal angle	0.978 (0.9573-0.9999)	0.049
>5 minutes to cannulate	Distal common bile duct diameter	0.940 (0.851-1.039)	0.228
>5 minutes to cannulate	Periampullary diverticulum	1.066 (0.202-5.627)	0.940
>1 unintended pancreatic duct cannulation/opacification	Choledochoduodenal angle	0.991 (0.968-1.014)	0.452
>1 unintended pancreatic duct cannulation/opacification	Distal common bile duct diameter	0.904 (0.799-1.022)	0.106
>1 unintended pancreatic duct cannulation/opacification	Periampullary diverticulum	1.397 (0.233-8.362)	0.715

MRCP predictors of the burden of difficult cannulation

In cumulative logit ordinal regression, both a narrower choledochoduodenal angle (OR 0.980, 95% CI 0.961-0.999; p = 0.040) and a smaller distal common bile duct diameter (OR 0.892, 95% CI 0.811-0.981; p = 0.019) were independently associated with a higher burden of difficult cannulation, defined by movement from zero to one to two to three fulfilled ESGE criteria. Periampullary diverticulum did not improve the model (OR 1.227, 95% CI 0.278-5.407; p = 0.787). These ordinal regression findings are also summarized in Table 2.

ERCP failure

MRCP anatomy alone was not associated with ERCP failure. In Firth penalized logistic regression, choledochoduodenal angle (OR 1.002, 95% CI 0.964-1.038; p = 0.924), distal common bile duct diameter (OR 0.980, 95% CI 0.794-1.147; p = 0.821), and periampullary diverticulum (OR 0.695, 95% CI 0.005-7.284; p = 0.806) were all non-significant.

Relationship between difficult cannulation burden and ERCP failure

**Figure 3:**
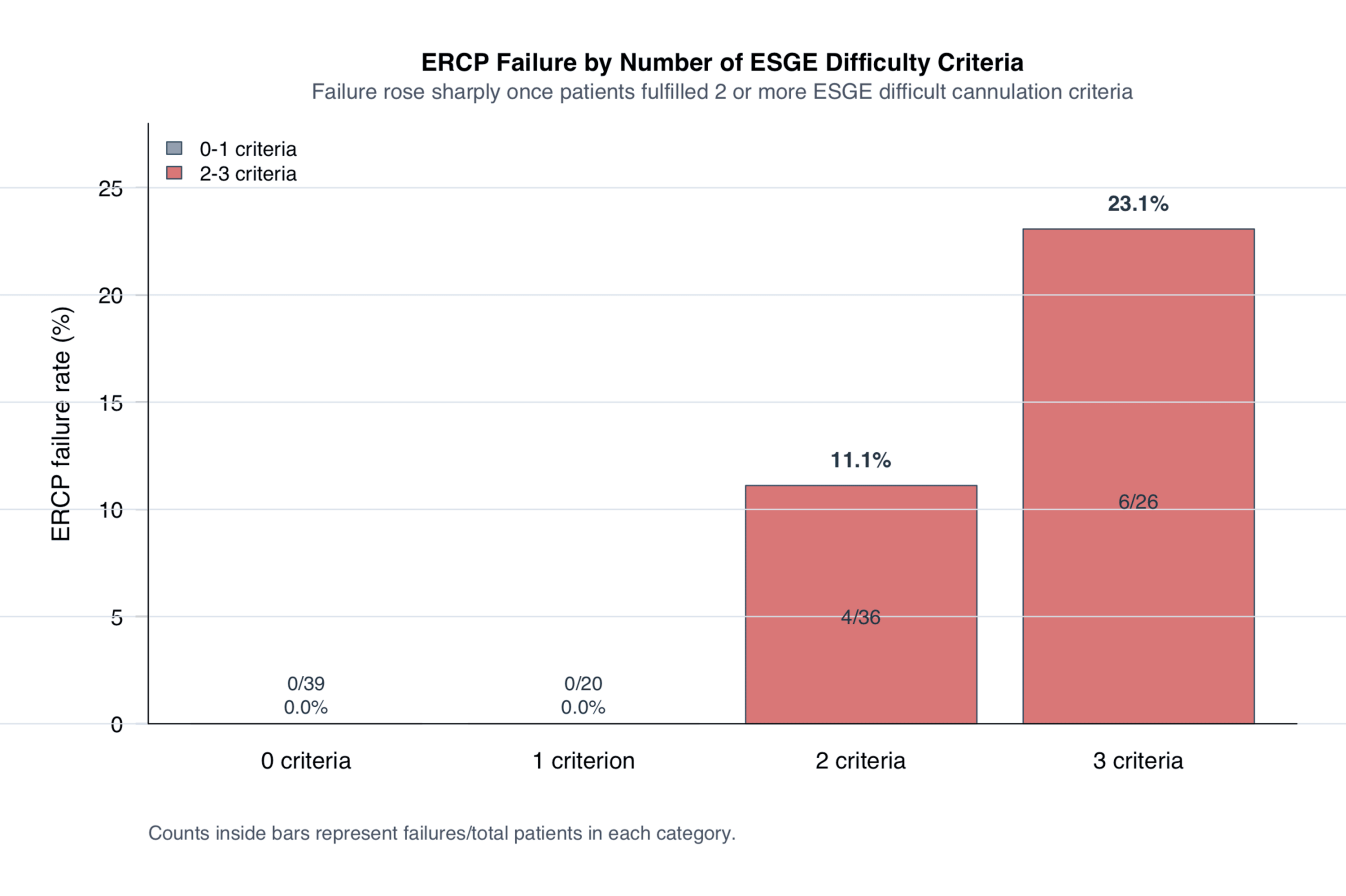
ERCP failure rate according to the cumulative number of ESGE difficult cannulation criteria met ERCP: endoscopic retrograde cholangiopancreatography; ESGE: European Society of Gastrointestinal Endoscopy

All ERCP failures occurred in patients with ESGE-defined difficult cannulation (10/82 vs 0/39, Fisher's exact p = 0.029). Failure was significantly associated with more than five papillary contacts (p = 0.0048) and with cannulation time greater than five minutes (p = 0.0017), whereas the association with unintended pancreatic duct cannulation or opacification did not reach statistical significance (p = 0.063). Failure rates increased stepwise with the number of ESGE criteria met: 0/39 for zero criteria, 0/20 for one criterion, 4/36 for two criteria, and 6/26 for three criteria. In Firth penalized logistic regression, each additional ESGE criterion increased the odds of ERCP failure by 3.66-fold (95% CI 1.67-11.39; p = 0.0003). Failure rates according to the number of ESGE criteria met are shown in Table 4 and Figure 3.

**Table 4:** Relationship between cumulative difficult cannulation burden and ERCP failure Each additional ESGE criterion increased the odds of ERCP failure by 3.66-fold (95% CI 1.67-11.39; p = 0.0003). ESGE: European Society of Gastrointestinal Endoscopy; ERCP: endoscopic retrograde cholangiopancreatography

Number of ESGE criteria met	Patients, n	ERCP failures, n	Failure rate
0	39	0	0.0%
1	20	0	0.0%
2	36	4	11.1%
3	26	6	23.1%

## Discussion

This study extends the literature on difficult biliary cannulation by focusing on MRCP-derived anatomy rather than on intraprocedural morphology alone. The most consistent anatomical signal across our analyses was a smaller distal common bile duct diameter, which independently predicted the global difficult-cannulation endpoint and remained relevant across the more granular difficulty models. By contrast, a narrower choledochoduodenal angle was less robust for the binary global endpoint but became more informative when the components and burden of difficulty were analyzed separately, suggesting that its effect may be most visible once cannulation becomes progressively challenging rather than simply dichotomized as easy or difficult.

The biological interpretation is plausible. Selective biliary access depends on the relationship between the papillary orifice, the intramural biliary segment, the axis of the distal bile duct, and the stability of the endoscopic approach. A smaller distal duct reduces the working target for a guidewire or sphincterotome, while an unfavorable choledochoduodenal angle may force a less stable or less coaxial approach during repeated attempts. This interpretation is concordant with the computed tomography findings reported by Lee et al. [27], is directionally supported by endosonographic work on duct-axis relationships [28], and is consistent with the broader endoscopic literature linking papillary shape and local anatomy to cannulation performance [16,17,20,24].

The relatively high frequency of difficult cannulation in the present cohort likely reflects the selected nature of a paired MRCP-ERCP population rather than an unselected ERCP population. In addition, the odds ratios for the anatomical variables should be interpreted in light of the measurement scale. Because they are expressed per 1-degree increase in choledochoduodenal angle and per 1-mm increase in distal common bile duct diameter, the apparent per-unit effect size is numerically small; however, these effects accumulate across clinically realistic anatomical differences and, therefore, remain meaningful in practice.

These associations should be interpreted cautiously. Several effect estimates were modest, and some confidence intervals approached the null value, while the study also included multiple related models without formal multiplicity adjustment. Accordingly, the present findings support an association between MRCP-derived anatomy and difficult cannulation, but they should be regarded as hypothesis-generating and require confirmation in larger, prospective external cohorts.

Our findings should therefore be interpreted as complementary rather than exclusive. Large multicenter and tertiary-center studies have shown that difficult cannulation is driven by a combination of papillary morphology, periampullary diverticulum, distal obstruction, inflammatory or malignant distortion, and operator-related factors [10,15-24]. MRCP does not replace those determinants, but it may contribute a meaningful pre-procedural geometric layer of risk information in patients who already undergo cross-sectional biliary imaging as part of routine care.

The indication profile of our cohort is also important for interpretation. Stone-related biliary disease was the leading indication, accounting for 84 of 121 ERCP procedures (69.4%), while smaller but clinically relevant subgroups underwent ERCP for malignant obstruction, infectious cholestasis, or stent-related reintervention. As a result, the present findings are likely most applicable to routine biliary ERCP in patients with distal obstruction or choledocholithiasis rather than to highly selected populations dominated by complex hilar strictures or postsurgical altered anatomy.

Periampullary diverticulum was not independently associated with difficult cannulation in our cohort. This negative result should be interpreted cautiously. Only seven patients had a recorded diverticulum, and our dataset did not distinguish between juxtapapillary and intradiverticular papillae. Contemporary evidence shows that the effect of diverticula on cannulation depends, in part, on the papillary relationship to the diverticulum and on evolving endoscopic expertise [18,19,25,26].

A particularly important result was that anatomy alone did not predict final ERCP failure, whereas the cumulative burden of difficult cannulation did. This supports the construct validity of the ESGE definition in our cohort and reinforces a key clinical concept: baseline anatomy may predispose to difficult access, but the intra-procedural trajectory ultimately determines whether the procedure fails. Once multiple ESGE difficulty criteria accumulate, the risk of failure rises sharply, and that stepwise pattern aligns with the rationale behind earlier rescue access and more protocolized stopping or escalation thresholds [5,6,10-14].

This contrast should also be interpreted cautiously. The anatomy-only failure model was based on only 10 failure events and was therefore underpowered, whereas cumulative ESGE difficulty burden is an intra-procedural measure that lies closer to the failure process itself. Thus, the stronger burden-failure association should not be interpreted as evidence that MRCP anatomy is unrelated to failure.

These observations may have practical implications. Awareness before ERCP of unfavorable distal biliary anatomy may help the endoscopist anticipate a higher likelihood of difficult access, prepare an earlier escalation strategy, and make more deliberate decisions about expert availability or rescue technique readiness. When that anatomical impression is followed by the accumulation of ESGE difficult cannulation criteria during the procedure, a more anticipatory pathway may be reasonable, including earlier senior operator involvement, timely rescue access, and individualized prophylactic planning against ERCP-related complications. Because complications were not uniformly captured in the current dataset, this clinical implication should be interpreted as hypothesis-generating rather than definitive.

The study also adds a practical element of novelty. Prior imaging-based work on cannulation difficulty has focused mainly on computed tomography or endoscopic ultrasound [27,28], whereas MRCP is already widely integrated into the routine evaluation of biliary obstruction and suspected choledocholithiasis. By translating anatomical risk stratification into MRCP, the present data speak to a modality that clinicians often already have in hand before therapeutic ERCP.

Several additional limitations should be emphasized. The study was retrospective, single-center, and modest in size, particularly for the ERCP failure endpoint. MRCP measurements were performed by a single radiologist blinded to ERCP outcomes, but formal interobserver and intraobserver reproducibility were not assessed. In addition, distal common bile duct diameter may partly reflect the underlying biliary pathology prompting ERCP, introducing potential confounding by indication, since the multivariable models were adjusted for MRCP anatomical variables rather than the full clinical context. Because only patients who underwent both MRCP and ERCP were included, selection bias toward more complex biliary disease is also possible. The manuscript also lacks representative, annotated MRCP images illustrating the measurement methodology, which may limit immediate radiological reproducibility for some readers. Finally, the observed association between cumulative ESGE difficulty burden and final ERCP failure should be interpreted cautiously, as these criteria are intra-procedural markers of cannulation struggle and therefore reflect procedural trajectory rather than a purely pre-procedural forecast.

Future studies should combine MRCP-derived anatomical measurements with papillary morphology, operator-level variables, and fully adjudicated adverse-event outcomes. Larger multicenter cohorts may also permit validation of formal pre-procedural risk scores, comparison with recently proposed cannulation difficulty models [24], and more advanced imaging approaches such as radiomics or semi-automated geometric modeling to determine whether MRCP-based risk stratification can be translated into a practical pre-ERCP planning or prophylaxis algorithm.

## Conclusions

MRCP-derived anatomy, particularly a smaller distal common bile duct diameter and a narrower choledochoduodenal angle, is associated with difficult ERCP cannulation and a greater burden of difficult cannulation. In contrast, final ERCP failure is more closely linked to the cumulative burden of difficult cannulation than to MRCP anatomy alone. These findings support the role of MRCP as a pre-procedural source of anatomic risk awareness in patients undergoing ERCP and suggest that imaging-based recognition of unfavorable duct anatomy may help inform earlier procedural planning in selected high-risk cases.
